# Solitary fibrous tumor of the pleura: biphasic contrast-enhanced CT findings with emphasis on differential diagnosis

**DOI:** 10.3389/fmed.2025.1604996

**Published:** 2025-05-27

**Authors:** Ying Xiao, Jiaer Chen, Sen Jiang, Ruowei Chen, Yangkang Li

**Affiliations:** ^1^Department of Radiology, Cancer Hospital of Shantou University Medical College, Shantou, China; ^2^Department of Medical Imaging, The Second Affiliated Hospital of Shantou University Medical College, Shantou, China

**Keywords:** solitary fibrous tumor, pleura, computed tomography, differential diagnosis, soft tissue tumor

## Abstract

**Objective:**

Solitary fibrous tumor of the pleura (SFTP) is a rare neoplasm. Familiarity of its radiologic features may allow preoperative diagnosis and improve management of patients. However, radiological studies on the comparison of imaging findings between SFTP and other thoracic tumors are scarce. This study aims to evaluate the radiologic features of SFTP on biphasic contrast-enhanced computed tomography (CT) images with focus on the differential diagnosis.

**Methods:**

The clinical data and CT images of 25 patients with pathologically proven SFTP and 42 patients with other types of thoracic tumors were retrospectively reviewed. Patient age, CT features including lesion size, shape, margin, precontrast density, intratumoral calcification, intratumoral vessel, enhancement degree, and blood supply were evaluated. Qualitative data were compared with Chi-square test and quantitative data were compared with *t*-test. When the radiologic features appeared to be significant in the univariate analysis, multivariate analysis was performed for SFTP group using logistic regression model. The diagnostic performance was established using the area under the receiver operating characteristic (ROC) curve.

**Results:**

Five CT features, including tumor size, contour, intratumoral vessels, marked enhancement, and blood supply from pulmonary circulation of two groups differed significantly (*P* < 0.05). Moreover, blood supply from pulmonary circulation was identified as the independent signs of SFTP by multivariate logistic regression analysis. The area under the ROC curve was 0.744 (*P* < 0.05).

**Conclusion:**

Solitary fibrous tumors of the pleura are often seen as large, well circumscribed masses with intense heterogeneous enhancement and multiple intratumoral vessels on CT images. For large tumors which are difficult to identify, carefully looking for the feeding artery from pulmonary circulation will be helpful to diagnose SFTP.

## Introduction

Solitary fibrous tumor of the pleura (SFTP), is a rare tumor that occurs in the pleura of mesenchymal origin, from the visceral pleura in about 80% (1). Most patients with SFTP are asymptomatic at the time of being diagnosed incidentally by routine chest radiography. Due to their non-characteristic clinical picture, SFTPs are usually diagnosed in the later stages of the development, when causing mass effect on the adjacent organs on account of their size (2). Complete enbloc resection of the tumor is the mainstay of therapy for SFTP. Despite the relatively benign disease course, questions remain open in the field of preoperative diagnosis and final treatment. The key points of therapy are to determine whether surgery is necessary and to ensure perioperative safety. The difficulty of surgery depends on the volume of the tumor, poor exposure, prominent blood supply, and pleural adhesion (3, 4). So knowledge of its radiologic features is very important and provides valuable data in preoperative diagnosis and surgical management of patients.

It is difficult to distinguish SFTP from other types of thoracic tumors due to lack of specific clinical symptoms and signs. SFTP may produce a large spectrum of imaging findings according to the descriptions in radiological literatures (5–7). Nevertheless, to the best of our knowledge, these studies were based on SFTP alone and lacked a comparative study with other thoracic tumors. Because the above radiological findings can also be found in many other types of thoracic tumors. Thus, how to improve the differential diagnosis accuracy between SFTP and other types of thoracic tumors is a problem need be solved.

Multislice spiral computed tomography (CT) with biphasic enhanced scan and 3D technology can not only demonstrate the enhancement features of SFTP but also provide valuable data on the exact location of tumor, important blood vessels, tumor heterogeneity, relationship to surrounding structures, and the presence of pleural effusion. In the present study, we aimed to describe the radiologic features of SFTP on CT images and provide a pertinent analysis on the differential diagnosis of this tumor. Knowledge of the key imaging features may help distinguish SFTP from other types of thoracic tumors, and enable the surgeon to anticipate SFTP preoperatively so that adequate procedures can be planned.

## Materials and methods

This study was approved by the Ethics Committee of our institution (Ethical Committee No. 2024054, 20 September 2024). In view of the retrospective study design, the need for informed consent was waived. All of the procedures were performed in accordance with the principles of the 1975 Helsinki Declaration and its later amendments (or with comparable ethical standards).

### Patients

Between January 2010 and December 2023, 34 patients with pathologically proven intrathoracic SFTP were identified. Inclusion criteria: (1) each patient did not receive any treatment; (2) the patient had complete CT data (plain scan and biphasic contrast-enhanced scan of thoracic area); and (3) the tumor was completely removed and confirmed pleural origin by surgery. Thus, a total of 25 tumors were selected in the SFTP group. The imaging database of our hospital was reviewed so as to identify control patients presenting in the same period with pathologically proven non-SFTP thoracic tumors. Inclusion criteria: (1) each patient did not receive any treatment; (2) the patient had complete CT data (plain scan and biphasic contrast-enhanced scan of thoracic area); and (3) the lesion demonstrated as a solitary mass without lymphadenopathy and metastasis. Finally, a total of 42 masses were evaluated in the non-SFTP group.

### CT examination and image analysis

Chest CT scans of 32 patients were performed on a 64-detector-row scanner (Philips Medical Systems, Cleveland, OH, United States), 35 patients were examined using a 16-detector-row scanner (GE Healthcare, Milwaukee, WI, United States). After unenhanced scan, a total of 75–100 ml contrast agent (Ultravist 300; Bayer Schering Pharma, Berlin-Wedding, Germany) was injected intravenously at a rate of 2.5 to 3.5 ml/s using a dual-barrel power injector. Saline flushes were administered after contrast bolus at the same injection rate. The arterial-phase scan was automatically triggered when the CT value of the aortic arch reached or exceeded 100 HU, and then the venous-phase scans were performed at interval of 60 s. Contiguous axial tomographic images, multiplanar reconstructions (MPRs) and maximal intensity projections (MIPs) were obtained. The slice thickness and interval were both of 5 mm. The reconstruction interval was 1.25 mm. All CT images were reviewed through a picture archiving and communication system (PACS). The images were replayed in mediastinum (*W* = 300, *L* = 30) and lung (*W* = 1200, *L* = −700).

All images were reviewed by two experienced radiologists (S.J., with 15 years of experience in oncological imaging, and W.B.Y., with 12 years of experience in oncological imaging). They did not know the clinical histories, laboratory tests, the results of the surgical resection, and pathological diagnoses of all patients. Image analyses were performed jointly and by consensus. The following signs of each lesion were evaluated: tumor size, shape, contour, density, calcification, intratumoral vessels, and degree of enhancement.

Specially, the size was measured using the longest diameter of the maximal MPR section as criteria. The shape was described as oval or irregular. The contour was depicted as distinct or indistinct. The density was classified as homogeneous and heterogeneous. Intratumoral vessels were evaluated on postcontrast images of the arterial phase. Moreover, post-contrast MIP images were also used to evaluate this sign. The degree of enhancement was assessed on postcontrast images of the venous phase and was subjectively classified as follows: mild, when the enhancement was similar to that of adjacent muscle; moderate, when the enhancement was higher than that of muscle, but lower than that of blood vessels; and marked, when the enhancement was approaching that of blood vessels.

The feeding artery of the lesion were also analyzed. Especially, post-contrast axial MPR images and 3D volume rendered (VR) images were used to evaluate this sign besides conventional images. For 3D VR images, CT angiographies only containing vascular anatomy were obtained using specific software (Autobone Removal). The software is dedicated to the automatic segmentation of bones from thoracic CT angiography data. Other anatomic structures were removed from the thorax of interest using volume-punching operations.

### Statistical analysis

The SPSS software package (version 26.0) was used for the statistical tests. Qualitative data were compared with Chi-square test and quantitative data were compared with *t-*test. *P* values of <0.05 were considered to be statistically significant. When the radiologic features appeared to be significant in the univariate analysis, multivariate analysis was performed for SFTP group using logistic regression model. The diagnostic performance of each feature was established using the area under the receiver operating characteristic (ROC) curve.

## Results

### SFTP group

There were 12 males and 13 females with a male to female ratio of 1:1.1 and an age of 19–83 years. The median age of diagnosis was 57.8 ± 14.8 years.

**FIGURE 1:**
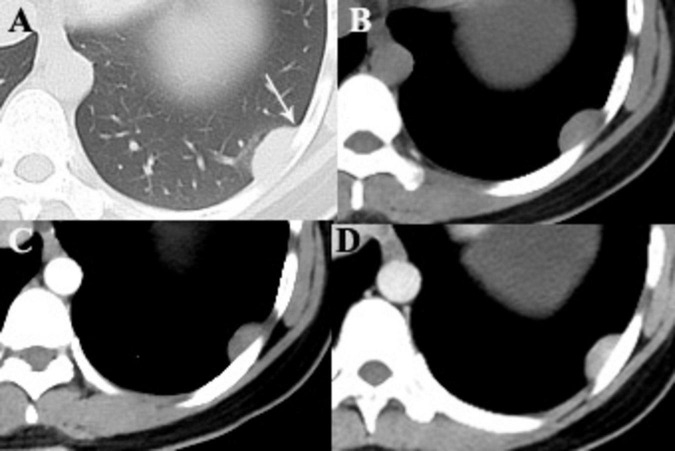
A 28-year-old woman with SFTP. **(A,B)** Precontrast CT scans demonstrate a small well-defined mass with homogeneous density in the lower left thorax. The mass forms an obtuse angle with the adjacent pleural surface (arrow). **(C)** No intratumoral vessels are detected in the arterial phase on postcontrast CT image. **(D)** Obvious enhancement is seen in the venous phase.

**FIGURE 2:**
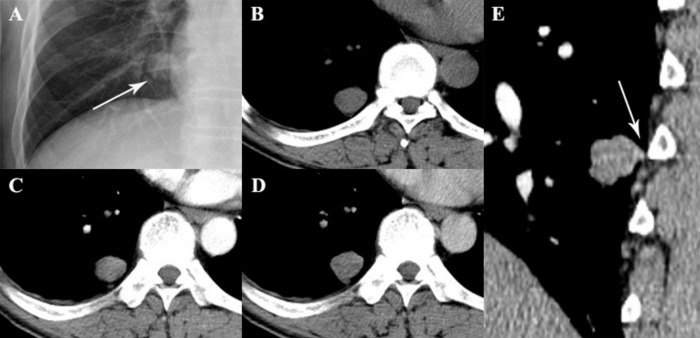
A 49-year-old woman with SFTP. **(A,B)** Chest radiograph and precontrast CT scan show a small oval mass with well-defined margin in lower right thorax (arrow). **(C,D)** The mass shows mild enhancement on biphasic postcontrast CT images. **(E)** A pedicle of the tumor with the adjacent pleura is detected on sagittal MPR image (arrow).

**FIGURE 3:**
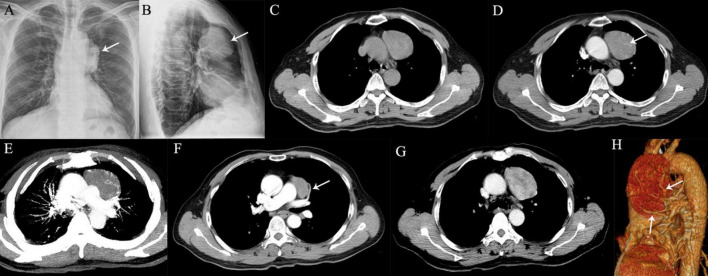
A 64-year-old man with SFTP. **(A,B)** Chest radiographs show a well-defined, homogeneous density opacity projecting the anterior left hilum (arrows). It was misdiagnosed as a mediastinal mass preoperatively. **(C)** Precontrast CT scans demonstrated an oval mass with well-delineated margin abutting the mediastinal surface. **(D)** Enlarged intratumoral vessels are seen in the arterial phase on post-contrast CT image (arrow). **(E)** The intratumoral vessels are depicted more clearer on MIP image. **(F)** A thick feeding artery arising from the left upper pulmonary artery is also detected (arrow). **(G)** Heterogeneous moderate to marked enhancement is detected in the venous phase. **(H)** Several feeding arteries arising from the left upper pulmonary artery to the tumor are showed on 3D VR image (arrows).

**FIGURE 4:**
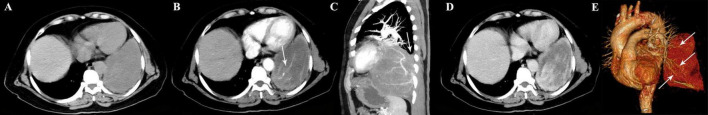
A 65-year-old woman with SFTP. **(A)** Precontrast CT scan shows a large mass in the left lower thoracic cavity. **(B)** Abundant intratumoral vessels are seen in the arterial phase image (arrow). **(C)** The intratumoral vessels are depicted more clearer on sagittal MIP image. Although the mass forms an acute angle with the pleura layers, a smooth tapering margin (arrow) is also seen. **(D)** The tumor shows heterogeneous marked enhancement in the venous phase. **(E)** Multiple feeding arteries arising from the left lower pulmonary artery to the tumor are showed on 3D VR image (arrows).

A total of 25 tumors were analyzed. Fifteen tumors located in the left inferior hemithorax, four in the left superior hemithorax, four in the right inferior hemithorax, and two simultaneously occupying the left superior and inferior hemithorax. The size of the tumors ranged from 2.0 to 22.5 cm (mean, 10.1 ± 5.5 cm). The shape was seen as oval (*n* = 13) or irregular (*n* = 12). All tumors showed a distinct contour. Calcification was seen in four tumors. On unenhanced CT images, the density was homogeneous (*n* = 6) or heterogeneous (*n* = 19). After contrast medium administration, abundant intratumoral vessels were seen in 15 cases in the arterial phase. In the venous phase, there were moderate to marked enhancement in 13, mild to moderate enhancement in 8, and mild enhancement in 4. One or more anomalous feeding arteries arising from pulmonary artery to the tumor were identified in 14 cases. The arteries branched into a spiderlike series of vessels on the surface of the tumor. A small amount of pleural effusion was seen in five cases. The characteristic CT findings of four SFTPs are depicted in Figures 1–4.

All 25 cases underwent surgery. Intraoperatively, all tumors were completely removed and were confirmed to originate from the pleura. Characteristically, the tumors were predominantly solid with complete capsule, tough texture, clear boundary, abundant blood supply, and gray-to-red or gray-to-white cross-section. Microscopic examination showed that tumors were composed of bland spindle-shaped cells and dense collagenous bands, with fascicular, irregular, or haphazard arrangements. A hemangiopericytoma-like vascular pattern with areas of hemorrhage and necrosis on the cut sections were seen in large masses. Cystoid spaces filled with mucoid material were also detected. Malignant SFTP was diagnosed if >4 mitoses/10 HPF were present, which was present in eight cases. Immunohistochemical staining revealed that the positive rate of CD34 was 92% (23/25), 64% (16/25) for CD99, 60% (15/25) for Vimentin, 52% (13/25) for BCL-2, and 28% (7/25) for Ki-67. Especially, the Ki-67 index was analyzed as the percentage of positive cells with nuclear staining in average of five high power field. The score was considered to be positive if the expression was equal to or greater than 10% and negative if the expression was less than 10%. STAT-6 staining was evaluated in 10 tumors and the positive rate was 100%.

### Non-SFTP group

There were 20 males and 22 females with a male to female ratio of 1:1.1. The median age of diagnosis was 56.1 ± 14.9 years for the total cases with a range of 18–86 years. Twenty-eight cases underwent needle biopsy, and 19 cases underwent surgery. A total of 42 masses were analyzed, including 5 solitary pleural mesotheliomas, 2 solitary pleural metastatic tumors, 2 pleural tuberculomas, 10 intrathoracic sarcomas, 3 lung cancers, 9 anterior mediastinal tumors (4 thymic tumors, 3 germ cell tumors, and 2 lymphomas), 10 posterior mediastinal tumors (9 schwannomas and 1 hemangioma), and 1 intercostal schwannoma in the lower left chest wall.

**FIGURE 5:**
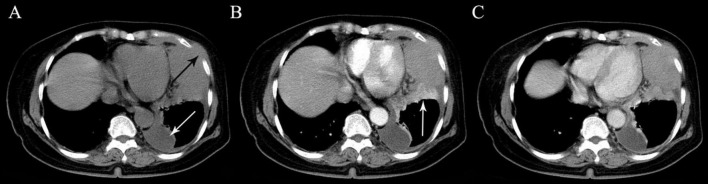
A 71-year-old woman with malignant pleural mesothelioma. **(A)** A solid mass with irregular shape is seen in the left lower thoracic cavity on precontrast CT. Intercostal soft tissue invasion (black arrow) and pleural effusion (white arrow) are also seen. **(B)** Segmental atelectasis with marked enhancement in the lower lobe of the left lung is detected in the arterial phase (arrow). **(C)** The tumor shows heterogeneous mild to moderate enhancement in the venous phase.

**FIGURE 6:**
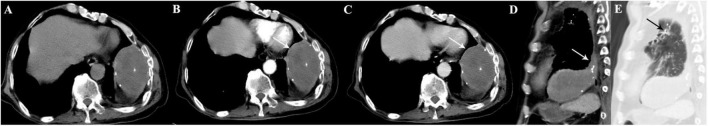
A 70-year-old man with pleural tuberculoma. **(A)** A large heterogeneous mass with multiple calcifications is seen in the left lower thoracic cavity on precontrast image. **(B,C)** On biphasic postcontrast images, the mass demonstrates peripheral mild enhancement (arrows), whereas no enhancement is observed inside the mass. **(D)** A tapering margin is seen on sagittal MPR image (arrow). **(E)** Concomitant obsolete pulmonary tuberculosis is also detected (arrow).

**FIGURE 7:**
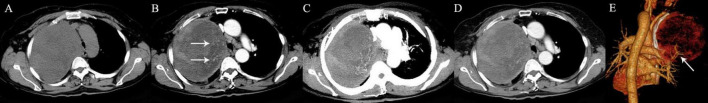
A 59-year-old woman with pulmonary undifferentiated pleomorphic sarcoma. **(A)** An oval mass is seen in the right upper lung. **(B)** Multiple intratumoral vessels are detected in the arterial phase (arrows). **(C)** The intratumoral vessels are depicted more clearer on MIP image. **(D)** The tumor shows heterogeneous mild to moderate enhancement in the venous phase. **(E)** Feeding arteries arising from the right upper pulmonary artery to the tumor are detected on 3D VR image (arrow).

**FIGURE 8:**
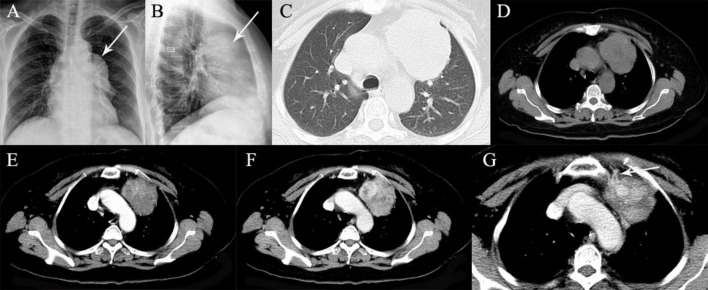
A 60-year-old woman with thymoma. **(A,B)** Chest radiographs show a well-defined opacity projecting the anterior left hilum (arrows). **(C,D)** Precontrast CT scans demonstrated a left anterior mediastinal mass with well-delineated margin. **(E,F)** On biphasic postcontrast images, the mass shows gradual enhancement and heterogeneous marked enhancement in the venous phase. **(G)** Feeding arteries arising from the left internal thoracic artery are detected (arrow).

**FIGURE 9:**
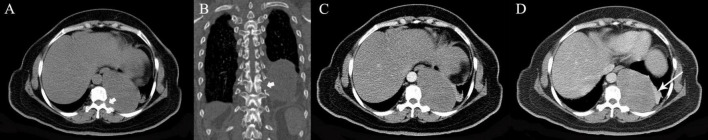
A 52-year-old woman with schwannoma. **(A,B)** Precontrast CT scans show a large mass in the left lower thoracic cavity involving the costovertebral recess (arrow heads). **(C,D)** On biphasic postcontrast images, the mass shows gradual mild to morderate enhancement. Segmental atelectasis with marked enhancement in the lower lobe of the left lung is detected (arrow).

The size of all lesions ranged from 1.9 to 15.0 cm (mean, 7.4 cm). The shape was seen as oval (*n* = 20) or irregular (*n* = 22). The contour was seen as distinct (*n* = 36) or indistinct (*n* = 6). On unenhanced images, the density was homogeneous (*n* = 5) or heterogeneous (*n* = 37). After contrast medium administration, multiple intratumoral vessels were seen in six cases in the arterial phase. In the venous phase, there was mild to moderate enhancement in 19, moderate to marked enhancement in 9, and mild enhancement in 14. Calcification was seen in 11 tumors. Blood supply from pulmonary circulation was seen in three cases. Blood supply from collateral internal thoracic artery was seen in one case. Pleural effusion was seen in 15 cases. The characteristic CT findings of five non-SFTP masses are depicted in Figures 5–9.

### Statistical results

Patient age of two groups did not differ significantly (*P* = 0.620). For the comparison of CT features, tumor shape, precontrast density, intratumoral calcification, and pleural effusion of two groups did not differ significantly (*P* > 0.05). Five CT features, including tumor size, tumor contour, marked enhancement, intratumoral vessels, and blood supply from pulmonary circulation of two groups differed significantly (*P* < 0.05). The statistical results of univariate analysis are summarized in Table 1.

**TABLE 1:** Statistical analysis of CT features between two groups.

CT features	SFTP	Non-SFTP	*P* value
Diameter (max, cm)	10.1 ± 5.5	7.4 ± 3.8	0.041*
Shape			0.729
Oval	13	20	
Irregular	12	22	
Contour			0.049*
Distinct	25	36	
Indistinct	0	6	
Density			0.791
Homogeneous	6	5	
Heterogeneous	19	37	
Calcification			0.506
Present	4	11	
Absent	21	31	
Intratumoral vessels			0.000*
Present	15	6	
Absent	10	36	
Marked enhancement			0.010*
Present	13	9	
Absent	12	33	
Blood supply from PC			0.000*
Present	14	3	
Absent	11	39	
Pleural effusion			0.174
Present	5	15	
Absent	20	27	

PC, pulmonary circulation.

*P < 0.05.

According to multivariate analysis, one separate sign independently associated with SFTPs was identified: blood supply from pulmonary circulation (Odds ratio = 21.636; 95% confidence interval, 4.239–110.444; *P* = 0.000). The area under the ROC curve was 0.744 (*P* = 0.001).

## Discussion

Solitary fibrous tumor of the pleura is a rare tumor of the thorax and comprises less than 5% of pleural tumors (8, 9). In the present study, SFTPs were found to have a peak incidence in the fifth decade of life, and affect both sexes equally, in agreement with the previous literature (10). Most of the tumors, whether benign or malignant, were large, oval masses with a well-circumscribed margin on CT images, which may be associated with the origin and the non-characteristic clinical picture of this kind of tumor. SFTP originate in mesenchymal cells not in the epithelium. They tend to grow in an expansive centripetal fashion and cause pressure on neighboring structures on account of their size. Adjacent organs are usually displaced, rather than invaded.

In the current study, most SFTPs were located in lower part of thoracic cavity, consistent to previous literatures (3, 5). Moreover, we found that the left lower thoracic cavity is the most common location in about 60% for this type of tumor. On CT images, small tumors exhibited a homogeneous soft-tissue mass which abutted the pleural surface and formed an obtuse angle against adjacent pleura surface, whereas, large SFTPs depicted a heterogeneous mass and formed an acute angle with the pleura surface. A pedunculated tumor or a “smoothly tapering angle” of the tumor with the adjacent pleura were considered two highly characteristic findings that could help in establishing the pleural location of the masses (6, 7).

A pattern of heterogeneous moderate to marked enhancement was present in 52% of tumors in this study. This sign was depicted more clearly in the venous phase after contrast administration. It indicates that a progressive enhancement is the enhancing pattern of this tumor. The term “geographic enhancement” was used to depict the characteristic of SFTP on postcontrast images (6). Good enhancement reveals the hypervascularity of the tumor parenchyma. Heterogeneity corresponds to intratumoral collagen and fibroblast, sareas of mucoid material, intralesional hemorrhage, necrosis or cystic change. This was confirmed on cut sections and microscopic examinations. In our opinion, large tumor size may outgrow tumor blood supply and cause intratumoral hemorrhage, necrosis or cystic degeneration. Furthermore, the hypervascularity of the tumor parenchyma may be the other reason to cause intralesional hemorrhage.

The other useful characteristic imaging finding of SFTP was prominent intratumoral vessels on post-contrast CT images, which also indicated the hypervascularity of the tumor. This finding was consistent to previous studies. Wignall et al. (7) reported that massive dilated intratumoral vessels and avid contrast enhancement were observed in 22 of the 34 tumors on post-contrast CT images. Cardinale et al. (6) also reported numerous intralesional vessels in approximately one fourth of their cases. This sign was observed more clearly in the arterial phase after contrast administration. In our series, it was detected in 15 SFTPs (60%) and was only seen in 6 tumors in the comparative group. So it can be considered as a valuable CT feature in diagnosing SFTP. Intratumoral vessels of SFTP usually have dilated lumen and marked enhancement. This classical imaging finding may be a result of increased vascularity and the formation of small vascular aneurysms in the tumor (11).

In the present study, a distinctive imaging feature of SFTP is the presence of collateral feeding vessel arising from the pulmonary artery, which was seen in 14 cases (56%). We found that the SFTPs had this sign were all larger tumors with the size ranging from 7.6 to 22.5 cm (mean, 13.6 cm). This sign was also present in 1 neuroendocrine carcinoma and 2 of 10 pulmonary sarcomas. In our experience, although it is not specific, this sign is helpful when present and can aid the radiologist in narrowing the differential diagnosis. The blood supply to the parietal pleura is provided by systemic vessels, whereas the visceral pleura is perfused predominantly by the pulmonary circulation (12). The blood supply from the pulmonary circulation reveals that SFTPs usually develop from the visceral pleura, more rarely from the parietal pleura (4, 9, 13). This was confirmed by operation in our study. Besides helping the radiologist to diagnose SFTP, another potential benefit of this imaging feature is in planning for embolization or ligation of vessels before surgery, which minimizes the risk of hemorrhage.

Due to the origin of pleura, firstly, it is necessary to differentiate SFTP from other lesions originating from the pleura, such as pleural mesothelioma, isolated pleural metastasis and pleural tuberculoma. Pleural mesothelioma usually develops from the parietal pleura and is associated with exposure to asbestos (14). Malignant pleural mesothelioma commonly shows a pleural mass or diffuse pleural thickening with peripheral invasion, and is often accompanied by massive pleural effusion. Benign pleural mesothelioma may mimic SFTP on unenhanced CT, however, a history of asbestos exposure and mild enhancement with the absent of pulmonary circulation supply are distinguished from SFTP. Isolated pleural metastasis is uncommon, especially that with hypervascularity. CT findings include pleural effusion, pleural nodule and limited pleural thickening. Diagnosis of the primary lesion allows it to be differentiated from SFTP. Pleural tuberculoma is an important sequelae of tuberculous pleurisy (15, 16). In the case of our study, CT examination demonstrated a large, oval mass with heterogeneous density, similar to a SFTP. However, low density on plain scan, multiple calcification and peripheral mild enhancement were detected, which were consistent to previous reports (15, 17) and were different from those obtained in SFTPs. Multiple intralesional coagulation, caseation necrosis, calcification, and scar tissue may be the reason for the lack of enhancement inside the tuberculoma (17).

When SFTP arises from a pulmonary fissure, it can appear to be surrounded by lung and simulate an intraparenchymal mass. Thus, intrapulmonary tumors include peripheral lung cancer or pulmonary sarcoma, and are necessary to distinguish from SFTP. Imaging findings described for peripheral lung cancer are lobular, short burr masses with pleural indentation (18, 19). These imaging findings are consistent to the cases in our study and are different from SFTPs. Intrathoracic sarcoma, including pulmonary sarcoma, usually shows a large, oval and heterogeneous mass, mimicking a SFTP. For some sarcoma types, obvious enhancement with intratumoral vessels can also be detected. It is quite difficult to differentiate these tumors from SFTP only according to these signs. Thus, carefully looking for the feeding artery from pulmonary circulation is helpful for differential diagnosis.

When SFTP has a mediastinal pleural origin and abuts the mediastinal pleural surface, it can mimic a mediastinal neoplasm. Thymic neoplasms, germ cell tumors, or lymphomas need be considered as differential diagnoses. In such cases, analysis of the mediastinum structures is fundamental. A true mediastinal mass usually expands, compressing the pulmonary parenchyma without causing mediastinal shift. Nevertheless, for lesions of pleural origin, the mediastinum is compressed and dislocated. Radiologically, typical thymoma is usually well-defined, round, or lobulated, homogenous and enhanced after contrast injection (20). However, it can be heterogeneous, or even cystic because of areas of hemorrhage and necrosis (21). Germ cell tumors usually occur in adolescence or early adulthood. Benign teratoma often exhibits a well-defined mass with calcification, fatty tissue and cystic areas. A large, irregular, ill-defined mass with peripheral invasion and varying degrees of enhancement is usually found in malignant germ cell tumors. Furthermore, serum tumor markers can help to differentiate them from other types of tumors. For cases that are difficult to distinguish, accurate evaluation of the blood supply may help to detect the origin of tumor.

When SFTP involves the costovertebral recess, it can mimic a peripheral neurogenic tumor. Neurogenic tumors are the most common type of tumor in this area, and the schwannomas constitute the largest group with a frequency over 50% (22). In our study, imaging findings described for schwannomas were oval, well-defined masses with predominantly cystic or solid cystic nature, in agreement with the literature reports (23, 24). The solid portion showed gradually enhancement and the area of cystic degeneration showed peripheral enhancement on post-contrast images. These findings are considered to be the typical features of this type of tumor and are different from SFTP. In this study, we also reported a case of cavernous hemangioma near the costovertebral recess. Thoracic hemangioma is rare, especially in posterior mediastinum. The lesion was unclear with adjacent pleura and was misdiagnosed as a SFTP preoperatively. Lack of calcification was not helpful in narrowing the diagnosis. However, like hepatic hemangioma, centripetal enhancement may be a clue to establish the diagnosis.

There are several limitations in our study. Firstly, the study sample size was small. Secondly, different CT equipments and techniques were used. Thirdly, MRI was absent in the evaluation of tumor. Forthly, a detailed pathological study was absent to be correlated to the imaging findings. Thus, further studies with expanded samples are required in the future.

## Conclusion

Solitary fibrous tumors of the pleura are often seen as large, relative high density masses with moderate to marked heterogeneous enhancement and abundant intratumoral vessels on CT images. For large tumors which are difficult to identify, carefully looking for the feeding artery from pulmonary circulation on post-contrast CT will be helpful to diagnose SFTP.

## Data Availability

The original contributions presented in this study are included in this article/supplementary material, further inquiries can be directed to the corresponding author.
